# *EGFR* transcription in non‐small‐cell lung cancer tumours can be revealed in ctDNA by cell‐free chromatin immunoprecipitation (cfChIP)

**DOI:** 10.1002/1878-0261.13093

**Published:** 2021-09-18

**Authors:** Christoffer Trier Månsson, Johan Vad‐Nielsen, Peter Meldgaard, Anders Lade Nielsen, Boe Sandahl Sorensen

**Affiliations:** ^1^ Department of Biomedicine Aarhus University Denmark; ^2^ Department of Oncology Aarhus University Hospital Denmark; ^3^ Department of Clinical Biochemistry Aarhus University Hospital Denmark

**Keywords:** cell‐free‐DNA, ChIP, circulating tumour DNA, diagnostics, EGFR, liquid biopsy

## Abstract

Determination of tumour‐specific transcription based on liquid biopsies possesses a large diagnostic and prognostic potential in non‐small cell lung cancer (NSCLC). Cell‐free DNA (cfDNA) packed in nucleosomes mirrors the histone modification profiles present in the cells of origin. H3 lysine 36 trimethylation (H3K36me3)‐modified nucleosomes are associated with active genes, and therefore, cell‐free chromatin immunoprecipitation (cfChIP) of H3K36me3‐associated cfDNA has the potential to delineate whether transcription of a particular gene is occurring in the cells from which its cfDNA originates. We hypothesized that cfChIP can delineate transcriptional status of genes harbouring somatic cancer mutations and analysed the recurrently observed *EGFR‐L858R* mutation as an example. In representative NSCLC cell lines, the relationship between wild‐type (WT) and mutated *EGFR* transcriptional activity and mRNA expression levels was analysed using H3K36me3 ChIP and *EGFR* mRNA reverse transcription quantitative PCR (RT‐qPCR), respectively. The ChIP analysis showed that both WT and mutated *EGFR* are transcribed and that mRNA is similarly expressed per *EGFR* copy. Based on this observation, we proceeded with *EGFR* cfChIP using blood plasma from NSCLC patients harbouring the *EGFR‐L858R* mutation. *EGFR‐WT* fragments can originate from both nontumour cells with no or low *EGFR* transcription and tumour cells with active *EGFR* transcription, whereas *EGFR‐L858R* fragments must specifically originate from tumour cells. H3K36me3 cfChIP followed by droplet digital PCR (ddPCR) revealed significantly higher enrichment of *EGFR‐L858R* compared to *EGFR‐WT* fragments. This is in alignment with *EGFR‐L858R* being actively transcribed in the NSCLC tumour cells. This study is proof‐of‐principle that cfChIP can be used to identify tumour‐specific transcriptional activity of mutated alleles, which can expand the utility of liquid biopsy‐based cfDNA analyses to enhance tumour diagnostics and therapeutics.

## Graphical abstract

Cell‐free chromatin immunoprecipitation (cfChIP) is a novel technique that can be used to investigate tumour gene expression in liquid biopsies. Up to our knowledge, this is the first study evaluating tumour gene activity based on cfChIP enriched mutated alleles. Here, we focussed on the recurrently observed EGFR‐L858R mutation in non‐small cell lung cancer and observed EGFR‐L858R displayed higher cfChIP enrichment than EGFR‐WT. Our research demonstrates that cfChIP has the potential to expand the utility of liquid biopsies in cancer.

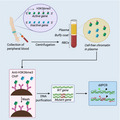

**Abbreviations**
cfChIP: cell‐free chromatin Immunoprecipitation
cfDNA: cell‐free DNA
ctDNA: circulating tumour DNA
ddPCR: droplet digital PCR
EGFR: epidermal growth factor
Ex19del: exon 19 deletion
GOI: gene of interest
H3K36me3: H3 lysine 36 trimethylation
MAF: mutational allele fraction
NSCLC: non‐small cell lung cancer
RT-qPCR: reverse transcription quantitative PCR
SCLC: small cell lung cancer
TKI: tyrosine kinase inhibitor
WT: wild‐type

## 1 Introduction

Lung cancer is the most common cause of cancer‐related death with an estimated 1.6 million deaths worldwide each year [1]. Lung cancer is divided into non‐small cell lung cancer (NSCLC) and small cell lung cancer (SCLC) based on histological phenotype, with NSCLC contributing to about 85% of all cases [2]. Sensitizing mutations in the epidermal growth factor gene (*EGFR*) such as exon 19 deletions (*ex19del*) and *L858R* mutations happen in about 15% of all NSCLC cases [3]. Acquisition of these mutations enables treatment with tyrosine kinase inhibitors (TKIs) such as erlotinib and osimertinib, which has improved overall survival [4, 5]. Resistance can occur via new somatic mutations in *EGFR* or through bypass mutations in other genes. Although identification of these bypass mutations is promising, the relevance of each mutation remains elusive. The new mutations are only relevant if expressed in the tumour.

Identification of *EGFR* mutations is done with tissue biopsies, but these can be misleading because of the well‐known heterogeneity of NSCLC tumours [6]. Liquid biopsies have been utilized to monitor acquired resistance in NSCLC patients showing promising results [7, 8, 9, 10, 11]. The use of liquid biopsies, such as blood samples, can give a greater insight into the cancer genetics [12]. In cancer patients, a fraction of the cell‐free DNA (cfDNA) in blood is circulating tumour DNA (ctDNA) that represents the genetics of the primary tumour as well as metastatic sites [13]. Furthermore, liquid biopsies allow longitudinal sampling with minimal risks for the patient compared to surgical biopsies and enable physicians to monitor treatment responses over time [14]. Monitoring responses is important during TKI treatment because eventually all NSCLC patients will develop TKI resistance, which leads to disease progression [15].

**Fig. 1:**
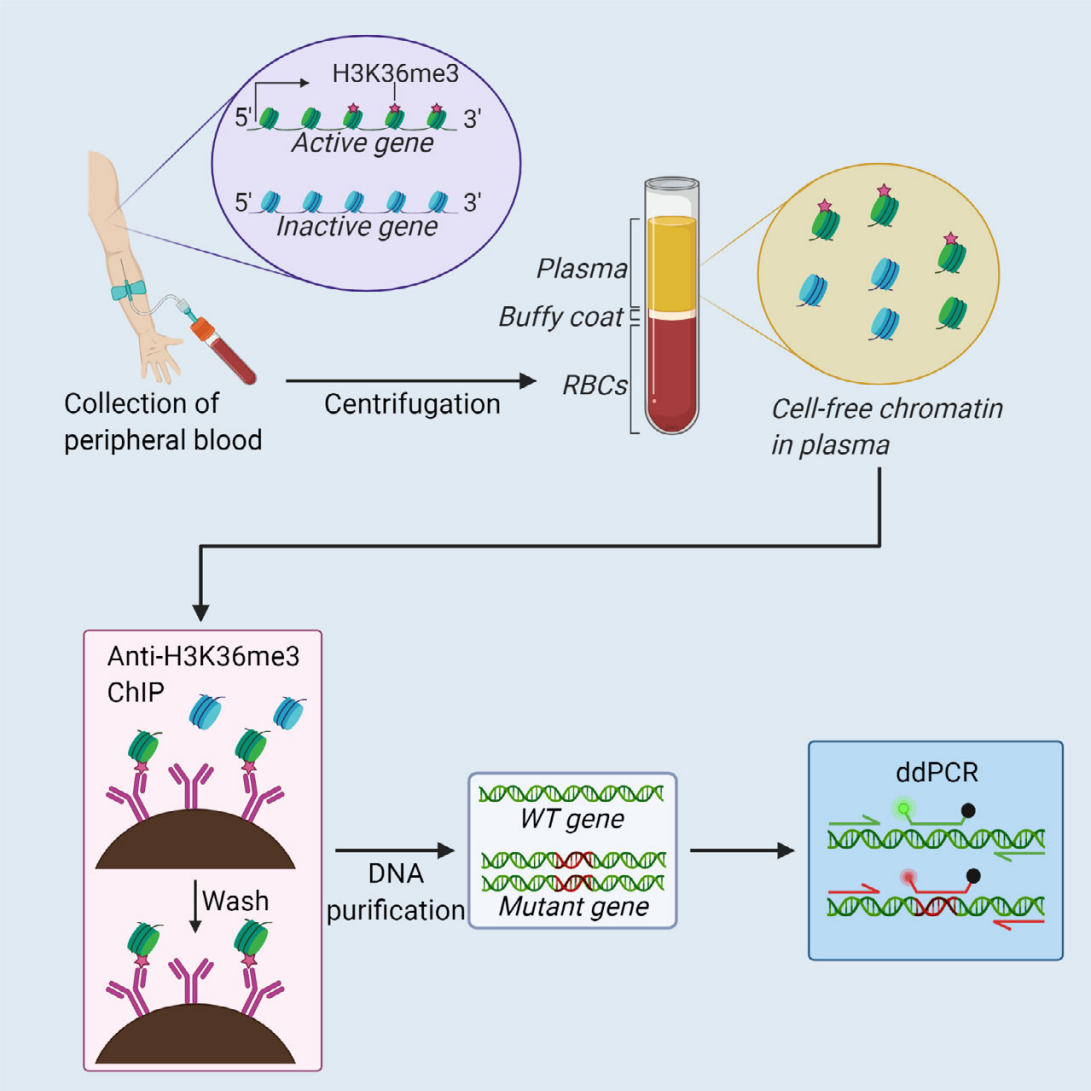
The H3K36me3 modification can be used to evaluate tumour gene expression with cell‐free chromatin immunoprecipitation (cfChIP). Active genes contain H3K36me3 in the 3′ end of the gene. Blood from lung cancer patients is separated by centrifugation, plasma is isolated, and H3K36me3‐containing nucleosomes are isolated with anti‐H3K36me3 chromatin immunoprecipitation (ChIP). Mutant and WT gene fragments are quantified using multiplex ddPCR. Red blood cells (RBCs), wild‐type (WT). Created with BioRender.com.

The recently developed cell‐free chromatin immunoprecipitation (cfChIP) technique published by our group [16] differentiates between transcriptional active and inactive gene fragments in cfDNA from plasma samples (Fig. 1). Histone H3 lysine 36 trimethylation (H3K36me3) is an epigenetic marker associated with nucleosomes located in the 3’ end of actively transcribed genes [17, 18]. Trimethylation of H3K36 is mediated by the histone methyltransferase SETD2 which is recruited to the phosphorylated C‐terminal of RNA polymerase II during gene transcription [19]. Furthermore, the level of H3K36me3 at a locus correlates with RNA abundance [18]. Therefore, measuring H3K36me3 levels in a gene can be used as an approximation of the transcriptional activity.

The H3K36me3 marker can be implemented on cfDNA to pull down fragments corresponding to transcribed genes. Thus, cfChIP can be used to determine from blood plasma whether a gene of interest (GOI) is transcribed in NSCLC tumours. As proof‐of‐principle, cfChIP was shown to have capability to distinguish NSCLC patients with adenocarcinoma from patients with squamous cell carcinoma based on a *KRT6* analysis [16]. Moreover, Sadeh *et al*. [20] presented the cfChIP‐seq method, which identifies transcriptionally active genes by next‐generation sequencing. In addition, the study demonstrates cfChIP‐seq can identify the transcriptional profile of the various tissues contributing to the cfDNA pool [20].

Here, we address whether blood plasma cfChIP can detect if genes harbouring somatic mutations indeed are transcribed in NSCLC tumours. For this, we examined a recurrently observed *EGFR* mutation in NSCLC. Based on NSCLC cell line experiments, we showed correspondence between wild‐type (WT) and mutated *EGFR* transcription measured with ChIP and mRNA expression. Furthermore, blood plasma cfChIP results demonstrate active transcription of *EGFR‐L858R* in NSCLC tumours. Thus, cfChIP enables detection of tumour‐specific transcriptional activity of genes harbouring somatic mutations.

## 2 Materials and methods

### 2.1 Blood plasma

This study was performed in accordance with the Declaration of Helsinki and accepted by the Central Denmark Region Committee on Biomedical Research Ethics (No. 1‐16‐02‐211‐16). Informed consent was obtained from all individuals. All patients had stage IV NSCLC and harboured an *EGFR‐L858R* mutation, which was verified with a tissue biopsy. All blood samples were collected before the patients received TKI therapy. Data of the NSCLC patients used in this study can be found in Table S1. Peripheral blood was collected from patients into EDTA tubes and centrifuged within 2 h at 1400 g for 15 min at room temperature. The plasma was aliquoted, kept at −80 °C and thawed on ice before it was applied to cfChIP.

### 2.2 Cell culture

H1975 (CRL‐5908, ATCC, LCG standards, Wesel, Germany), HCC827 (CRL‐2868, ATCC, LCG standards, Wesel, Germany) and A549 (CCL‐185, ATCC, LCG standards, Wesel, Germany) cells were grown in RPMI medium containing 10% fetal calf serum and 1% penicillin‐streptomycin (Gibco, Thermo Fischer Scientific, Waltham, MA, USA). The cells were cultivated at 37 °C in 5% CO_2_.

### 2.3 cDNA synthesis

RNA was extracted from the cells using TRI Reagent according to the manufacturer’s instructions (Sigma‐Aldrich, St. Louis, MO, USA). The RNA concentration was measured using NanoDrop One (Thermo Fischer Scientific, Waltham, MA, USA), and 1 µg RNA was subjected to cDNA synthesis. cDNA was synthesized using IScript cDNA Synthesis Kit (Bio‐Rad, Hercules, CA, USA) according to the manufacturer’s instructions.

### 2.4 ChIP

Conventional ChIP from cultured cancer cells (approximately 1.5 × 10^6^ cells) was done with anti‐H3K36me3 IgG (Abcam, ab9050) as described [16].

cfChIP from blood plasma was done with anti‐H3K36me3 IgG (Abcam, ab9050) as described [16]. Briefly, between 200 and 400 µL plasma was aliquoted from each sample and the extracted DNA used as input sample. The rest of the plasma sample (0.6–2.9 mL) was diluted to a ratio of 1 : 5 in RIPA buffer (Tris/HCl 25 mm, NaCl 150 mm, Sodium deoxycholate 1%, NP‐40 1%, SDS 0.1%) containing EDTA‐free cOmplete‐brand protease inhibitor (Roche, Mannheim, Germany) and used for cfChIP.

### 2.5 Quantitative PCR (qPCR)

qPCR experiments were run in triplicates of 10 µL containing forward and reverse primer (1.25 µm), 5 µL RealQ 2X Master Mix green with ROX (Ampliqon, Odense, Denmark), and 3.75 µL RNase‐free water. qPCR was performed with Roche LightCycler 480 with the following settings: 15 min at 95 °C, 40 cycles of PCR (10 s at 95 °C, 20 s at 58 °C and 15 s at 72 °C). This step was followed by a final elongation of 1 min at 72 °C. All Ct values > 35 were considered below threshold. The data were quantified using the X_0_ method [21]. Primer sequences and primer efficiencies, determined as previously described [21], can be found in Table S2. In RT‐qPCR analyses using EGFR primers for exon 6 and 7, the data were normalized to *ACTG1* as previously described [16].

### 2.6 Droplet digital PCR

Droplet digital PCR (ddPCR) experiments were run in duplicates using the QX200 AutoDG Droplet Digital PCR System (Bio‐Rad). Each reaction contained 11 µL ddPCR Supermix for Probes (no UTP), 1 µL forward and reverse primer (10 µm), 1 µL of each probe (1 µm), 5–10 µL cDNA or ChIP sample and nuclease‐free H_2_O to a total volume of 22 µL. For cell input and ChIP samples, equal amounts of DNA (0.35 ng) were added to each well. The cfChIP ddPCR experiments were made using the ddPCR mutation detection assay EGFR‐L858R (Bio‐Rad, Catalogue No. 10049550, ID dHsaMDV2010021). The baseline for a positive sample has been determined by ddPCR analysis of cfDNA from 14 healthy individuals. 23 315 *EGFR* fragments were detected (avg. 1665 per sample), and all of them were *EGFR‐WT*, and none were *EGFR‐L858R*. This means that no false positives are produced by the assay. Droplets were made using the QX200 AutoDG (Bio‐Rad). Semi‐Skirted ddPCR plates (Bio‐Rad) were sealed using PX1 PCR Plate Sealer (Bio‐Rad), and PCR was performed using a GeneAmp PCR System 9700 (Applied Biosystems, Waltham, MA, USA). Droplets were read using the QX200 Droplet Reader (Bio‐Rad), and the data were analysed in QX Manager 1.1 Standard Edition. Positive droplets were normalized to the total number of droplets generated. Template‐free controls with nuclease‐free H_2_O instead of cfChIP, cDNA or ChIP samples were used to set the threshold for positive droplets. In gene copy number analysis, *ALK*, which is expected to be present in two gene copies per cell, is used for normalization. Primers and probes are listed in Tables S2 and S3.

### 2.7 Statistics

Statistic results for gene expression were calculated using data from independent biological replicates. Comparison of statistical significance was performed using a ratio‐paired Student’s *t*‐test. A two‐sided *P*‐value < 0.05 was considered statistically significant.

## 3 Results and Discussion

### 3.1 Anti‐H3K36me3 ChIP on NSCLC cell lines reveal EGFR transcription

We first addressed the relation between *EGFR* transcriptional activity measured with H3K36me3 ChIP and *EGFR* mRNA expression levels measured by quantitative cDNA analyses in representative NSCLC cell lines. We note H3K36me3 is a marker for transcriptional activity, and this histone modification is expected to be present in the 3′ end of an actively transcribed gene [19]. We included in our analyses HCC827 harbouring allelic amplification with approximately 70 copies of the *EGFR* deletion, *EGFR‐ex19del*(c.2236_2250del15); H1975 with 4 to 5 *EGFR* copies, due to aneuploidy of chromosomes carrying the heterozygous missense mutation *EGFR‐L858R* (c.2573 t > g); and A549 with 2 copies of WT *EGFR* [22, 23, 24, 25, 26]. To differentiate between WT and mutated *EGFR*, we utilized ddPCR, which allows multiplex absolute quantification. We designed mutation‐ and WT‐specific *EGFR* primer and probe sets for ddPCR analysis of ChIP and cDNA samples. This included a probe conjugated to HEX specifically targeting *EGFR‐ex19*(c.2236_2250del15) and a FAM‐conjugated probe binding to *EGFR‐ex19WT* discriminating between WT and mutated *EGFR‐ex19* in HCC827 cells [27]. Similarly, we designed a HEX‐conjugated probe targeting *EGFR‐ex21*(c. 2573 t > g) and a FAM‐conjugated probe binding to *EGFR‐ex21WT* discriminating between *EGFR‐WT* and *EGFR‐L858R* present in H1975 cells [28].

**Fig. 2:**
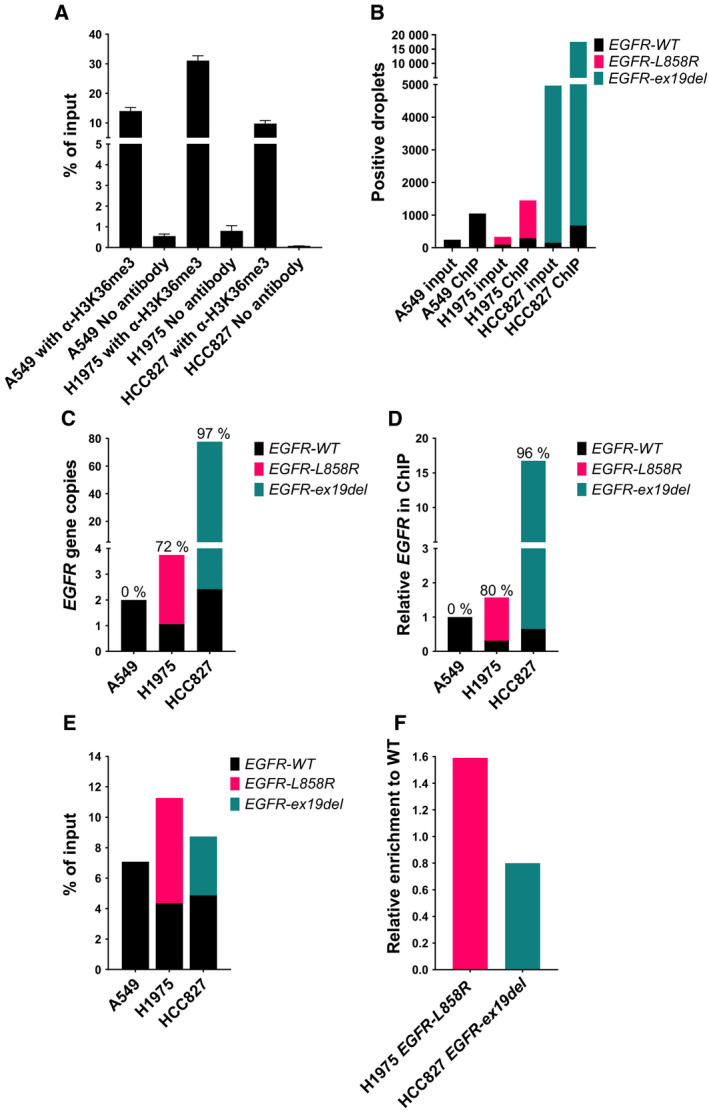
H3K36me3 chromatin immunoprecipitation (ChIP) analysis in NSCLC cell lines of WT and mutated *EGFR*. (A) ChIP enrichment of *EGFR* measured with conventional qPCR. Results are shown as enrichment in percentage of input. Bars display the mean with the SD of 3 technical replicates. (B) The total number of *EGFR‐*WT, L858R and ex19del positive droplets in droplet digital (ddPCR) detected following ChIP. The data are normalized to the total amount of droplets generated for each sample. (C) Determination of *EGFR* gene copy numbers in input samples normalized to the gene copy number of *ALK*. The MAF is displayed above columns for mutated *EGFR* in HCC827 and H1975 cells. (D) Mutated and WT *EGFR* in ChIP samples, normalized to A549 *EGFR‐WT*. (E) The ChIP enrichment of *EGFR* shown as % of input. (F) Ratio of ChIP enrichment calculated as % of input for mutated *EGFR* relative to % of input for *EGFR‐WT* for HCC827 and H1975 cells. Experiments in diagram B–F are performed 3 times and the data displayed is a single representative experiment.

We performed ChIP with an H3K36me3 antibody using chromatin extracts from A549, H1975 and HCC827 cells. As a control, ChIP was performed without antibody added to the ChIP reaction. The specificity for H3K36me3 antibody‐mediated enrichment of *EGFR* DNA fragments was first verified by conventional qPCR using an amplicon located to *EGFR* exon 19 (Fig. 2A). We next determined the ChIP enrichment distribution of WT and mutated *EGFR* by ddPCR (Fig. 2B–F). The total number of *EGFR*‐positive droplets in input and ChIP samples is presented in Fig.␣2B. The results validate the specificity of the primer and probe sets, given *EGFR‐ex19del* is only detected in HCC827 cells and *EGFR‐L858R* is only detected in H1975 cells. As expected, input and the H3K36me3 ChIP‐enriched *EGFR* DNA is almost exclusively represented by *EGFR‐ex19del* in HCC827 cells (Fig. 2B). This is in accordance with the known *EGFR‐ex19del* amplification in HC827 cells. In H1975 cells, the input and H3K36me3 ChIP‐enriched *EGFR* DNA is preferentially represented by *EGFR‐L858R* (Fig. 2B).

H1975 has about 4 *EGFR* gene copies with *EGFR‐L858R* representing 72% of the *EGFR* genes (Fig. 2C). Following ChIP, the mutational allele fraction (MAF) increases, indicating higher *EGFR‐L858R* transcription compared to *EGFR‐WT* transcription in H1975 cells (Fig. 2D). *EGFR* amplification in HCC827 cells results in approximately 75 *EGFR* gene copies, with *EGFR‐ex19del* representing 97% of the alleles. The MAF following ChIP is similar to the MAF detected in input for HCC827 cells (Fig. 2C,D), which shows *EGFR‐ex19del* and *EGFR‐WT* alleles are transcribed to a similar level.

The normal presentation of ChIP results as enrichment of GOI (hereby *EGFR*), compared to input will represent the transcriptional activity per *EGFR* copy. The ChIP enrichment relative to input for WT and mutated *EGFR* is displayed in Fig. 2E. The ratio between ChIP‐enriched mutated and WT *EGFR* following H3K36me3 ChIP is shown in Fig. 2F. This shows no major difference exists in ChIP enrichment for WT versus mutated *EGFR* in the examined cell lines per gene copy and supports the idea that WT and mutated *EGFR* alleles are transcribed to a comparable level. Thus, the total number of *EGFR* alleles is the major determinant for the overall transcriptional level in the analysed cell lines.

### 3.2 NSCLC ChIP experiments verified with RT‐qPCR

**Fig. 3:**
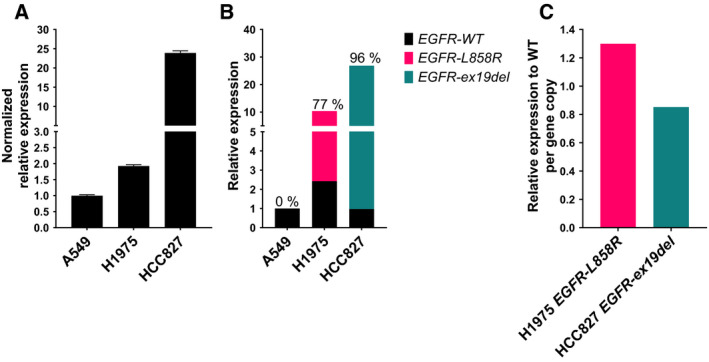
*EGFR* mRNA expression analyses in NSCLC cell lines. (A) *EGFR* mRNA expression was determined by reverse transcription quantitative PCR (RT‐qPCR) and presented relative to *ACTG1*. Bars display the mean with the SD of 3 technical replicates. (B) ddPCR‐based mRNA expression analysis of mutated and WT *EGFR*. Data are presented relative to the *EGFR‐*WT expression level in A459 cells. (C) ddPCR‐based mRNA expression analysis of mutated relative to WT *EGFR*. Data are presented normalized to gene copy numbers in H1975 and HCC827 cells. Experiments in diagram B and C are performed 3 times, and the data displayed are a single representative experiment.

We next proceeded to *EGFR* mRNA expression analyses. RT‐qPCR mRNA expression analyses with an *EGFR* amplicon targeting exon6 and exon7 showed that H1975 and HCC827 cells have approximately 2‐ and 24‐fold more *EGFR* mRNA expression compared to A549 cells, respectively (Fig. 3A). Given the *EGFR* copy number differences we previously described in A549 (2 copies), H1975 (4–5 copies), and HCC827 (75 copies), this points to *EGFR* mRNA expression levels and H3K36me3 ChIP enrichment levels showing a similar correlation to the *EGFR* copy number (Figs 2C and 3A). We next performed mRNA expression analyses using ddPCR to detect WT and mutated *EGFR* mRNA expression in the cell lines. From Fig. 3B, it can be seen that in both H1975 and HCC827 cells, mutated *EGFR* is expressed to a higher level than WT *EGFR*. Figure 3C shows the mRNA expression ratio between mutated and WT *EGFR*. To compare results from ChIP (percentage of input, Fig. 2E) with RNA expression, the gene copy number of *EGFR*‐*WT* and mutated *EGFR* must be taken into consideration. In Fig. 3C, the *EGFR* expression levels in Fig. 3B are normalized to *EGFR* copy numbers presented in Fig. 2C and then plotted relative to *EGFR‐WT* expression for H1975 and HCC827 cells. The RNA expression ratios for mRNA per gene copy (1.30 and 0.85 for H1975 and HCC827, respectively) are similar to the ChIP enrichment ratios (1.59 and 0.80 for H1975 and HCC827, respectively; Figs 2F and 3C). Thus, the results validate the correspondence between mRNA expression and H3K36me3 ChIP‐derived transcriptional results for *EGFR*. We concluded that in the examined NSCLC cell lines, H3K36me3 ChIP and quantitative cDNA analyses revealed WT and mutated *EGFR* per gene copy are similarly transcribed and expressed at the mRNA level and that these two different measures for gene expression are correlated for *EGFR*.

### 3.3 Tumour‐specific EGFR expression measured in ctDNA with cfChIP

**Fig. 4:**
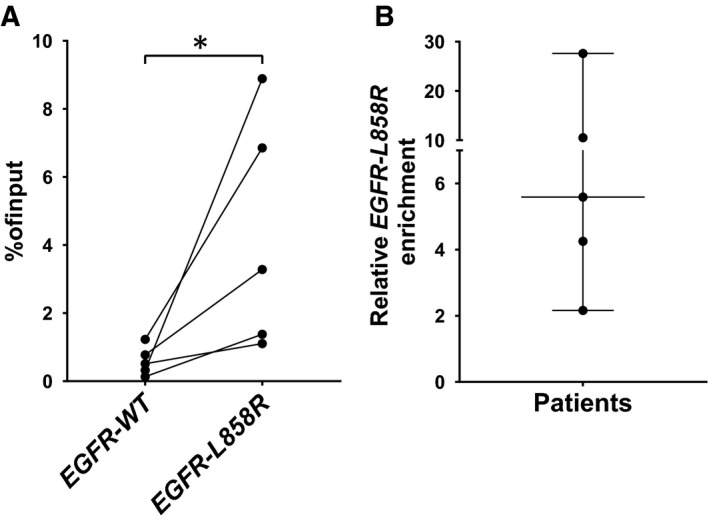
Cell‐free chromatin immunoprecipitation (cfChIP) can reveal tumour‐specific *EGFR‐L858R* transcription. (A) *EGFR‐WT* and *EGFR‐L858R* H3K36me3 cfChIP enrichment plotted as per cent of input. Results from the same patient are connected by lines (*n* = 5). (B) Relative enrichment of *EGFR‐L858R*. Enrichment is calculated by dividing the cfChIP enrichment in % of input of *EGFR‐L858R* by the cfChIP enrichment in % of input of *EGFR‐WT* for each blood plasma sample. Dots show each blood plasma sample, the line indicates the median, and error bars show ± the 95% CI (*n* = 5). Statistical significance was determined by a ratio‐paired Student’s *t*‐test. *P < 0.05.

We next performed H3K36me3 cfChIP using blood plasma from NSCLC patients harbouring *EGFR* mutations. For NSCLC patient blood plasma samples, all mutated *EGFR* fragments in the cfDNA pool will represent ctDNA. Given the expected driver status of mutated *EGFR*, most, if not all, of the mutated *EGFR* ctDNA fragments represent DNA expressed in the tumour cells at the time the gene was released to the blood as ctDNA. On the other hand, the WT *EGFR* fragments in the cfDNA pool can originate from any of the cells in the body delivering cfDNA to the blood, including tumour cells contributing *EGFR‐WT* ctDNA fragments, but WT *EGFR* will most often originate from normal tissue. Not all noncancerous *EGFR‐WT* fragment‐contributing cells have *EGFR* transcription to a level comparable to the tumour cells.␣This was demonstrated with *EGFR* expression being low in whole blood (data source: GTEx Analysis Release␣V8, dbGaP Accession phs000424.v8.p2, ENSG00000146648.17), which is the major contributor of cfDNA [29]. Thus, if mutated *EGFR* is transcribed in the tumour, then the H3K36me3 modification must be expected to occur more frequently at *EGFR‐L858R* mutated fragments relative to *EGFR‐WT* fragments in the pool of cfDNA. This is a different scenario from *EGFR* ChIP in NSCLC cell lines where WT and mutated *EGFR* always originate from the same cells. We focussed our blood plasma cfChIP analyses on patients harbouring the *EGFR‐L858R* mutation, which is present in about 40%–45% of all *EGFR‐*mutated NSCLC tumours [30]. Information on the included patient plasma samples is available in Table S1. The H3K36me3 cfChIP results are presented as enrichment in the percentage of input (Fig. 4A). The results showed enrichment of significantly more *EGFR‐L858R* fragments compared to *EGFR‐WT* fragments. This is in alignment with *EGFR‐L858R* indeed being transcribed in the tumour and proof‐of‐concept that cfChIP can determine whether a gene harbouring a somatic mutation is actively transcribed and accordingly tumour expressed. Figure 4B illustrates the considerable variation in the degree of *EGFR‐L858R* enrichment between NSCLC patient blood plasma samples (2‐ to 27‐fold). Besides reflecting differences in *EGFR‐L858R* transcription, enrichment can be affected by multiple factors, including variance in the amount of circulating cfDNA bound to nucleosomes, loss of histone methylation, dissociation of cfDNA from the nucleosome core particle [31], and loss of sample material during the cfChIP procedure. Understanding the variation in cfChIP enrichment within the same patient is highly relevant; however, due to the limited number of plasma samples taken pre‐EGFR‐TKI therapy for each patient we were not able to obtain such data. The cfChIP enrichment of *EGFR‐L858R* has a median of 3.3%, which is comparable with the efficiency obtained with conventional ChIP from cancer cell lines (Figs 2 and 4). The H3K36me3 antibody used for cfChIP limits the analyses to genes harbouring mutations in the 3′ region. Sadeh *et al*. [20] showed that targeting histone modifications at the promoter region of actively transcribed genes is also feasible. We used ddPCR to generate cfChIP results for GOIs, but next‐generation sequencing of cfChIP samples would in theory contain all expressed genes present in the cfDNA pool. This was proved by Sadeh *et al*., who showed that cfChIP‐seq can be used to determine the cell of origin as well as diagnose different diseases [20]. However, since the presented cfChIP‐seq design doesn't focus on tumour‐specific mutations, it is difficult to determine if a cfChIP‐seq enriched gene is expressed in the tumour, or it originates from healthy cells. Furthermore, since H3K4me3 is restricted to the transcription start site of active genes, H3K36me3 cfChIP‐seq, also presented by Sadeh *et al*., is envisaged to be superior regarding detection of transcriptional activity in genes with somatic mutations.

Our study adds to Sadeh *et al*. because we demonstrate that it is possible to use the cfDNA technique to look exclusively at the tumour‐derived ctDNA and not at the total pool of cfDNA. Focusing on somatic mutations present in the cancer can help in understanding the relevance of concomitant mutations in *EGFR*‐mutated patients. Blakely and colleagues [10] have shown that patients harbouring *EGFR* mutations also acquire multiple different alterations that could affect the patients’ TKI response. cfChIP could give a greater insight into the relevance of these mutations because only actively transcribed genes harbouring mutations should affect the TKI response.

## 4 Conclusion

cfChIP targeting somatic cancer mutations is a promising new methodology for which the basis of liquid biopsies could help understand how expression of mutated oncogenic drivers such as *EGFR*, *ALK*, *MET* and *KRAS* contribute to carcinogenesis. Liquid biopsies allow longitudinal sampling with minimal risks for the patient compared to surgical biopsies and enable monitoring of the dynamics in oncogenic driver expression during the cancer disease period. Further studies will help to increase the understanding of the utility of cfChIP and appropriate ways to implement the cfChIP method for improved cancer treatment and diagnosis.

## Conflict of interest

The authors declare no conflict of interest.

## Author contributions

CM, ALN and BSS conceived and designed the study. CM performed the experiments, analysed the data and drafted the manuscript. PM contributed with clinical data and patient material. JVN contributed with methodological input and discussions. All authors revised and approved the final manuscript.

## Supporting information

Table S1. Input and cfChIP sample data from patients with *EGFR‐L858R*.
Table S2. Sequence, primer efficiency, amplicon length, and application of primers.
Table S3. Sequence and tag on probes.

## Data Availability

All data required to evaluate the conclusions of the paper are present in the main text or the Supplementary Materials of the paper.
