# Supplementary material for: EGFR transcription in non‐small‐cell lung cancer tumours can be revealed in ctDNA by cell‐free chromatin immunoprecipitation (cfChIP)
Source: Mol Oncol. 2021 Sep 18;15(11):2868–76. doi: 10.1002/1878-0261.13093 (PMC8564636; doi:10.1002/1878-0261.13093)
Supplement: Supplementary file 1 — Table S1. Input and cfChIP sample data from patients with EGFR‐L858R. Table S2. Sequence, primer efficiency, amplicon length, and application of primers. Table S3. Sequence and tag on probes. [file MOL2-15-2868-s001.docx]

| Patient | EGFR  Status | cfDNA concentration  (ng/mL) | Input MAF (%) | cfChIP MAF (%) | cfDNA used for cfChIP (ng) | Positive droplets | | | |
| --- | --- | --- | --- | --- | --- | --- | --- | --- | --- |
|  |  |  |  |  |  | EGFR WT input | EGFR L858R input | EGFR WT cfChIP | EGFR L858R cfChIP |
| Patient H | L858R | 58.45 | 15 | 43 | 35.07 | 164 | 29 | 4 | 3 |
| Patient I | L858R | 59.15 | 29 | 47 | 59.15 | 367 | 153 | 9 | 8 |
| Patient J | L858R | 27.83 | 8 | 33 | 80.69 | 215 | 19 | 19 | 10 |
| Patient K | L858R | 187.25 | 6 | 39 | 524.30 | 3226 | 197 | 30 | 19 |
| Patient L | L858R | 71.75 | 3 | 50 | 100.45 | 155 | 6 | 4 | 4 |

Table S1.

| Name | Sequence (5’-3’) | Primer efficiency | Amplicon length | Application |
| --- | --- | --- | --- | --- |
| EGFR_ex19_fwd | CTCTGGATCCCAGAAGGTGAG | 75.54 % | 81 or 96 bp | ddPCR/qPCR |
| EGFR_ex19_rev | ATCGAGGATTTCCTTGTTGGCTT |  |  |  |
| EGFR_ex20_fwd | CGCCTGCTGGGCATCTG | 78.17 % | 96 bp | ddPCR |
| EGFR_ex20_rev | GTCTTTGTGTTCCCGGACATAGT |  |  |  |
| EGFR_ex21_fwd | CCGCAGCATGTCAAGATCAC | 94.43 % | 77 bp | ddPCR |
| EGFR_ex21_rev | CCTTCTGCATGGTATTCTTTCTCT |  |  |  |
| EGFR_ex6/7_fwd | ACTGCTGCCACAACCAGT | 73.71 % | 126 bp | RT-qPCR |
| EGFR_ex6/7_rev | GGGGTTGTAGAGCATGAGTG |  |  |  |

|  |  |
| --- | --- |

Table S2.

| Name | Sequence (5’-3’) |
| --- | --- |
| EGFR_ex19-WT | FAM-CGCTATCAAGGAATTAAGAGAAGCAACATCTCC-BHQ1 |
| EGFR_ex19-del15 | HEX-TAAAATTCCCGTCGCTATCAAGACATCTCC-BHQ1 |
| EGFR_ex21_WT | FAM-TTTGGGCTGGCCAAACT-MGBEQ |
| EGFR_ex21_L858R | HEX-TTTGGGCGGGCCAAACT-MGBEQ |

Table S3.
